# Silver Lining of Haze: The Mixed Effect of Haze on Donation

**DOI:** 10.3389/fpsyg.2020.02042

**Published:** 2020-08-26

**Authors:** Shihao Li, Guoqun Fu, Jingting Yuan, Jingyu Wu

**Affiliations:** ^1^Department of Marketing, Business School, University of International Business and Economics, Beijing, China; ^2^Department of Marketing, Guanghua School of Management, Peking University, Beijing, China; ^3^China Construction Bank, Beijing, China; ^4^Department of Economics, Faculty of Business and Economics, University of Hong Kong, Hong Kong, China

**Keywords:** haze, mortality salience, donate, time, money

## Abstract

Considering little literature investigate the influence of haze on humans in psychology field and the increasing frequency of haze hitting China, and its remarkably adverse impacts on society, this research uses two studies to explore the mixed effect of haze on donation behavior, and aims to make contribution to current literature and provide insights to haze issues. Study 1: 110 participants were included into a weather information survey in which half of them were instructed to read haze weather information, and the other half were assigned to good weather information condition. After reading and recalling experiences under the same weather condition, all participants were displayed and asked to report their attitudes on a donation program, including their donation intention with money and time, the amount of donating money, and the behavior measurement about whether they would like to leave their email addresses to the charity organization to keep in further connection. The results showed people in haze weather condition, compared with whom in good weather condition, were more likely to donate money and less likely to donate time to the donation program. There is a significant interaction effect between haze or not and donation type on donation intention. We did not find effect of haze on the amount of donation and donating behavior. Study 2: 101 participants were randomly assigned to haze weather condition or good weather condition first and then were asked to judge a donation program as study 1. After that, we measured mortality salience using three items as our mediator variable. The results showed there was a significant interaction effect between haze (vs. good) weather and donation type on donation intention which replicated the results of study 1. People in haze condition would donate more money and less time compared with people in good weather condition. Besides, we showed mortality salience was the underlying mechanism. People in haze condition perceived higher level of mortality salience, which altered their attitudes on money and time resources. Across two studies, we found convergent evidence supporting our hypotheses. Specially, haze weather can increase donation intention with money resources but decrease donation intention with time resources. This effect is mediated by mortality salience caused by haze. Based on our results, we conclude with an exploratory discussion.

## Introduction

In recent years, with the haze getting more and more frequent and severe, more attention has been paid to find the influence of haze on the society and human behavior. Biologists focus on the effect of haze on human bodies, and they found that haze may cause mental disorder and affect the secretion of neurotransmitters (Calderon-Garciduenas et al., 2015). Environmental science field researchers investigate the formation and evolution of haze weather (Wang et al., 2006; Huang et al., 2014). Sociologists investigate the cause, harm, public reaction, and countermeasures of haze (Wang and He, 2014). Limited research has been done in psychology field to investigate what consequences haze can bring to people. This paper tries to bridge this theoretical research gap and address the research question empirically by exploring how haze affects human decision-making and behavior. Specifically, we want to examine the influence of haze on donation and see whether there are some social benefits other than damage that haze can bring to us.

To answer the question, we have several propositions. First, as negative environment, haze may make people more emotionally aroused with respect to the pollution, thus they are more prone to put in money and more willing to allocate resources toward pollution abatement. As a consequence, haze will increase people’s donation intention. Second, because of remarkably adverse impacts on human health, we propose that death threat would be more salient in haze weather, and the mortality threat triggered by haze may value money less while it may value time more; therefore, the positive relationship between haze and donation is moderated by donation type. Specifically, compared with the control group, haze group has higher intention to donate money, but lower intention to donate time.

In this article, we first review the literature on pollution and its effects on donation intention. Then, we develop the hypothesis that haze may induce mortality salience and then increase people’s intention to donate money and decrease people’s intention to donate time. We examined this hypothesis in two studies. In study 1, we tested the moderation effect of donation type, and found participants in haze condition (vs. good weather condition) will be more likely to donate money and less likely to donate time to charity organization. In study 2, we examined the underlying mechanism of this effect. Our results revealed that people perceived more mortality threaten in haze condition (vs. good weather condition), and this mortality salience drove different valuations for money and time. Finally, we closed with a discussion of the contribution and the implications of this research for the broader topic of haze.

## Literature Review and Hypotheses

### Environment and Haze

The physical environment exerts an influence on people from several aspects to a different extent. Kinnafick and Thøgersen-Ntoumani (2014) research indicated that the physical environment and physical activity can independently improve positive affect. Steidle and Werth (2013) conducted four studies to demonstrate that both priming darkness and actual dim illumination improved creative performance. Stewart et al. (1983) raised a procedure based on judgments of human observers for measuring visual air quality in urban areas, and its reliability and validity are examined using the results of several studies conducted in a metropolitan area. Various physical environments influence on the risk perception of people and risk buying behavior (Yoon and Choi, 2016). Davison and Lawson (2006) research results highlight links between the physical environment and children’s physical activity. Viollon et al. (1998) developed an audio-visual approach for assessing the sound perception of the urban environment.

Negative environment, such as pollution, affects individual psychological response, for instance, emotion, affection, etc. Bullinger (1989) found that impairments in reaction time to visual stimuli and in ability to concentrate appeared to be associated with increased pollutant concentrations. Multiple time-series analyses revealed area-related effects of SO_2_ on mood and stress synchronously as well as with a time delay of 1–4 days. Zeidner and Shechter (1988) showed that high degree of anger and anxiety could be aroused by air pollution. Chattopadhyay et al. (1995) revealed that residents of the industrial area (with air pollution) were highly affected in terms of physical and mental health. They complained of throat and eye irritation, respiratory problems, tension, and anxiety much more than the inhabitants of the residential area (non-polluted area).

Negative environmental factors could also make a difference on individual behavior. A previous study revealed that perceived level of pollution is a stronger predictor of affective reactions and willingness to pay to reduce pollution than is the objective level of pollution (Zeidner and Shechter, 1988). Furthermore, individuals more emotionally aroused with respect to a polluted environment are more prone to put in time and more willing to allocate financial resources toward pollution abatement.

Haze, as a phenomenon of severe negative environment, exerts remarkable impacts on individuals physically and psychologically, including anxiety, depression, respiratory disease, cancer, and premature death (Wang and Mauzerall, 2006; Xu et al., 2013). Specifically, in the next section, we would elaborate that people think more about death in haze weather condition and this mortality salience could influence their attitudes on money and time.

### Haze and Mortality Salience

In a benign environment, mortality is not a conscious concern that is easy to be accessible in human memory. According to terror management theory (Greenberg et al., 1986), mortality salience means that individuals are more aware of the possibility of their own death, usually after reminding of death-related signals (Ferraro et al., 2005; Schindler et al., 2019). Researches show that stuff which is related to death can serve as reminders to make mortality more salient, such as diseases (White et al., 2013), funeral home (Pyszczynski et al., 1996), death-related media contexts (Liu and Smeesters, 2010), insurance brands or products (Fransen et al., 2008), natural disasters (Fritsche et al., 2010), etc. Even the subliminal prime that shows the word “death” or explicit manipulation such as writing the imagining essay about death can activate the mortality construct, which is referred to as mortality salience (Arndt et al., 1997; Hayes and Schimel, 2018; Chen et al., 2019).

There are two main antecedents of mortality threat that related to our research, which we would elaborate on these two factors. The first one is diseases (White et al., 2013; Hill et al., 2015). Researchers found out that when people perceived higher risk of diseases because of the heuristic cues of illness, such as pathogen load in environment, the concerns about disease would activate mortality salience, which would further lead to stress response corresponding to the threat (White et al., 2013; Hill et al., 2015). For example, women with a history of vulnerability to illness respond to these cues by desiring a greater number of novel partners (favor sexual variety seeking) than without such history (Hill et al., 2015). Second is the natural disaster. Previous literature argues that environmental events such as natural disaster can influence people’s affect, which in turn has an impact on an individual’s judgments (Västfjäll et al., 2013). Leiter (2011) also has shown that environmental disasters or fatal events such as avalanche can expose the individual to mortality risk. After directly seeing the consequences of the natural hazard (usually its life-taken consequences), individuals are more aware of the mortality. This article also reviews the attributes of the disaster that influences people’s perception of risk. Three basic attributes are as follows: (1) the number of people affected by a hazard; (2) voluntariness and controllability of risks; (3) familiarity with risks can impact people’s perception of the severity of the disaster. When the disaster affects many people, and people lose control of the consequences, as well as when people are familiar of the disaster, they tend to perceive higher risk of the disaster.

The field about haze has been an understudied area. We propose that haze would serve as a signal for mortality salience both because, as we argued earlier, it is the cue for disease and it is also a kind of pollution that related to environmental disaster. For one thing, in the haze day, we are exposed to high level of PM2.5 and more likely to get respiratory diseases. People who are more sensitive to air pollution would have severe physical symptoms. These potential risks of getting ill can activate the concept of death, which increase the mortality salience. For another, haze is one kind of natural hazard (Carn et al., 2009), even if it is caused by human behavior which can be called air pollution. It has some characteristics which increase the perceived risk of the pollution. For instance, exposure to haze is involuntary and inevitable, and only to some degree controllable (e.g., by migrating to less endangered living areas, using mouth-muffle and air cleaner). Besides, people are very familiar with bad influences of haze so people feel more risky to be faced with haze, which would increase mortality salience.

To sum up, we propose that haze would increase an individual’s mortality salience by serving as the reminder of disease and natural hazard.

H1: Haze increases individual’s mortality salience.

### Mortality Salience and Donation

Terror management theory (Sheldon et al., 2004), based on the body of work by cultural anthropologist Becker (1973), proposes that much of human social behavior is rooted in coping with the basic knowledge of human mortality. That is, mortality salience can lead to anxiety and existential terror. These momentary affective states would influence the process of judgment and may have many behavioral consequences (Schwarz and Clore, 1983), such as more increased intentions to engage in physical fitness activities, higher preference for luxury goods, and materialism (Ferraro et al., 2005). The literature shows that humans have developed anxiety buffer strategies to prevent existential terror, which can be divided into two behavior tendencies. On the one side, people have the basic striving for self-preservation resulting from fear of death, which usually has the manifest that people are more defensive or holding a more valid cultural worldview, stronger in-group bias (Zagefka and James, 2015). On the other hand, people that know about the inevitability of one’s own death are assumed to perceive themselves as living in accordance with the standards of this worldview (i.e., self-esteem), which may activate legacy motive and self-esteem constructed behaviors (Mandel and Smeesters, 2008). For example, people who are manipulated by death signal are more like to engage in pro-environment behavior and donate more money for the next generation (Wade-Benzoni et al., 2012). This is because being a valuable member of one’s own culture may allow the self to transcend beyond individual death and thus might provide a sense of symbolic immortality (Fritsche et al., 2010). Moreover, there are some papers suggesting that mortality salience can increase donations (Zagefka and James, 2015).

As we have summarized earlier, mortality salience could both increase the resource self-preservation, by being more defensive and conservative, and increase legacy motivation, by spending resources for the next generation and other people. So the core is that what is the moderator of these two conflict behavior tendencies? We propose that people with mortality salience are more likely to donate money but less likely to donate time.

### Donation Type: Money vs. Time

Time and money are two basic resources that can satisfy different needs and have different associations. There is little contention that these two basic resources have different effects on a variety of judgments and behaviors (Mogilner and Aaker, 2009). For example, time is associated with company by social groups (“spending time with families”), but money is associated with work (“hard work for money”). Researchers have shown that recalling a nostalgic event would cause participants to give away more money, but not time, than recalling an ordinary past life event. Nostalgia increases the motivation for time but decreases the motivation for money because nostalgia boosts the need for social connection, which can be satisfied with time but not money (Lasaleta et al., 2014). For another example, money is associated with unethical behavior while time is related to ethical incidence, so manipulating participants to focus on money instead of time could even make people less ethical, more hardworking, and less socially active (Gino and Mogilner, 2014). Concerning donation, different ways of asking (ask participants to donate money vs. time) can have different effects because asking for money activates more exchange mindset than asking for time. The results show that asking for time can increase the donation intention than asking for money (Liu and Aaker, 2008).

We propose that mortality salience can change people’s value or utility function about these two resources, money and time. Intuitively, after people know that death is inevitable, people would be more likely to devalue money because it is not related to symbolic meaning. For example, people who emphasize the hedonic experience would like to spend more money to fulfill their materialism needs (Kasser and Sheldon, 2000). As another example, people manipulated with mortality salience focus more on legacy motivation, such as donating money for pro-environmental campaign (Wade-Benzoni et al., 2012). Zagefka and James (2015) also find that awareness of one’s own mortality can increase money donations. Thus, we propose that mortality salience would increase people donation intention with money resources. However, when death signal is more salient, people are more aware of the scarcity of time (King et al., 2009), and the scarcity of objects can enhance their value (Dai et al., 2008). Besides, King et al. (2009) also suggested that reminders of death (the scarcity of time) should render time more valuable, so it is reasonable to infer when mortality is salient in people’s minds, people would perceive scarcity of their life and time, and then value time more, and thus less likely to donate time. Therefore, we propose that mortality salience can decrease the donation intention with time resources.

H2: Haze can increase donation intention with money resources while it can decrease donation intention with time resources.

H3: Haze can increase donation intention with money resources but decrease donation intention with time resources, which is mediated by mortality salience caused by haze.

## Experiment 1: The Impact of Haze on Donation Intention

Study 1 was an initial test of the hypotheses that haze can increase donation intention with money resources but decrease donation intention with time resources. We divided the haze group and control group by showing the participants with different weather information. Both conditions assigned participants to read a short context about a donation to save a student who got lung cancer. Our rationale was that participants who experienced the haze condition will be more willing to donate money after seeing the lung cancer report compared with those who experienced the good weather condition.

### Research Method

#### Participants and Design

Study 1 is a 2 (weather condition: haze vs. good) × 2 (donation intention type: money vs. time) mixed-subjects design, with donation intention type being the within-group factor. The dependent variables are the donation intention, the amount of donation, and behavioral measurement on donation (give email address or not). One hundred and ten participants (*M*_*age*_ = 30.23, SD = 8.34; 64.5% female) were recruited in this experiment.

#### Materials and Procedures

Participants received the survey online and learned that they would take part in two short and unrelated studies. First, participants took a weather perception survey and were asked to read the weather information about China of 1 day in the past. They were randomly assigned to haze condition or good weather condition. In the haze condition, the information emphasized the air quality index (API) was very bad and visibility was very low. Moreover, a picture of Tiananmen Square nearly disappearing in the heavy haze was followed by the information. In contrast, information in good weather condition emphasized the API was very good and visibility was very high. Besides, a picture of Tiananmen Square with blue sky also was shown next. All participants were asked to recall and write down their experience and feelings under similar weather conditions after reading the information.

The participants then took the donation survey by reading a news report about a donation program for people who got lung cancer. After reading this news report, participants were asked to answer four questions which served as our dependent variables. We will elaborate the details in the next subsection. Then, participants were asked to report their attitudes on weather condition in the first survey using one item “How do you think the pollution level in the first survey?” (1 = not at all, 7 = very severely) and their emotion “How about your emotion?” (1 = very negative, 7 = very positive). We collected the demographic information at the end of study, including gender, age, and monthly income.

#### Dependent Measures

In this part, participants were first asked to read a news report, which said that a student who studied in Beijing was diagnosed with lung cancer, and his family could not afford high medical expenses. China Love Foundation is launching a “Special Fund for Respiratory Diseases,” calling on the public to join the donation project to support that student. This news was followed by our dependent measurement, which included three parts. The first part was the measurement of donation intention on different donation types (money vs. time). We used two items to measure the extent of that participants would like to donate their money and time. These two items were: “To what extent are you willing to donate money to this organization” (1 = not at all, 7 = strongly); and “The Fund also needs volunteers to carry out some works such as activity planning which would take some time. To what extent are you willing to participate in these volunteer activities?” (1 = not at all, 7 = strongly).

The second and third parts of dependent measurement were to enrich our understanding about donation willingness. We measured the amount of money participants would like to donate. “If you have 100 RMB for free, how much are you willing to donate to this project?” (0–100 RMB). Besides, we also conducted behavioral measurement, “If you are willing to become such a volunteer which would take some time, please leave your E-mail, the foundation’s staff will contact you later on. If you are not, please skip it.” (write down the email address or not). Thus, we included donation intention (money and time), donation monetary amount, and behavioral measurement for time donation as our dependent variables.

### Data Analysis

Study 1 mainly investigated the impact of haze weather condition on donation, especially whether people in haze condition, compared with good weather condition, would like to donate more money and less time. Therefore, this study first conducts a MANOVA to analyze the main effect of haze on donation, and then we adopt a repeated measures ANOVA to examine the moderation effect of donation type.

### Results

#### Manipulation Checks

For the manipulation checks, we conducted a one-way ANOVA to test whether haze condition perceived the environment as more polluted. The analysis revealed the predicted effect that participants in the haze condition perceived the air shown in the picture as more polluted (*M* = 6.39) than the control condition (*M* = 3.05; *F*(1,108) = 165.74, *p* = 0.000, η^2^ = 0.61). This finding suggested that the haze manipulation was successful.

#### Hypothesis Testing

We first tested whether the haze manipulation affect respondents’ donation intention and behavior. A MANOVA was conducted and results showed that respondents in haze condition and control condition did not significantly differ in the amount of money they were willing to donate (*M*_*haze*_ = 39.33 vs. *M*_*good*_ = 32.58; *F*(1,108) = 1.63, *p* = 0.205). The two conditions showed significant differences at the intention to donate money (*M*_*haze*_ = 5.71 vs. *M*_*good*_ = 5.25; *F*(1,108) = 4.19, *p* = 0.043, η^2^ = 0.04) and the intention to donate time (*M*_*haze*_ = 4.67 vs. *M*_*good*_ = 5.19; *F*(1,108) = 4.07, *p* = 0.046, η^2^ = 0.04). The results remained the same when gender, age, income, and emotion were included into the analysis. Besides, we measured the behavioral measurement for time donation by coding the participant who wrote down the email address as “1” and otherwise as “0” and conducted χ^2^ test. The results did not show a significant difference between haze condition and good weather condition (χ^2^(1) = 1.59, *p* = 0.250). We then averaged the intention to donate money and the intention to donate time as the index of overall donation intention, and there was no main effect of haze on donation (*F*(1,108) = 0.03, *p* = 0.869).

**FIGURE 1:**
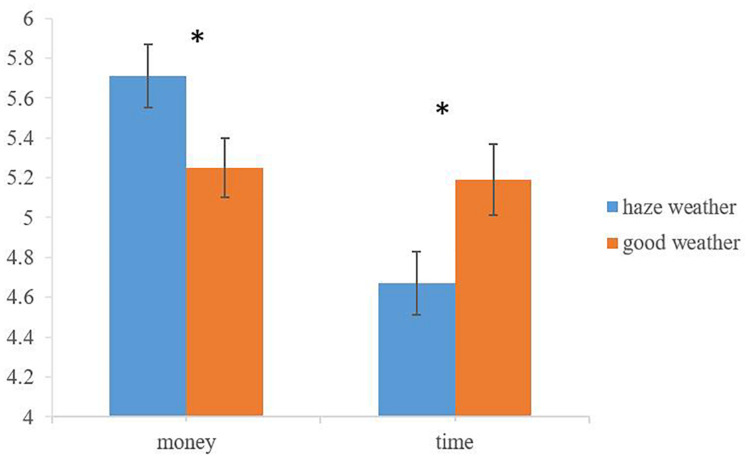
Moderation effect of donation type. *Significant at *p* < 0.05.

Next, we tested the moderation effect of donation type, that is the relationship between haze and donation is moderated by donation type. Specifically, compared with the control group, haze group has higher intention to donate money, but lower intention to donate time. We conducted a 2 (weather condition: haze vs. good) × 2 (donation type: money vs. time) repeated measures ANOVA with the second factor being within subjects. The analysis revealed a weather condition × donation type interaction (*F*(1,108) = 15.87, *p* < 0.001, η^2^ = 0.13; see Figure 1). Specifically, contrast analysis showed that people in haze condition were more likely to donate money (*M*_*haze*_ = 5.71 vs. *M*_*good*_ = 5.25; *F*(1,108) = 4.19, *p* = 0.043, η^2^ = 0.04) and less likely to donate time (*M*_*haze*_ = 4.67 vs. *M*_*good*_ = 5.19; *F*(1,108) = 4.07, *p* = 0.046, η^2^ = 0.04) than people in good weather condition. These findings supported the moderation effect of donation type on the relationship of haze on donation intention.

### Discussion

In study 1, we examined our hypotheses and got the initial support. Our data showed that if we averaged the donation intention on money and time, there was no main effect of haze on the averaged donation intention. This result indicated that the effect of haze on donation intention was only validated by donation type (money vs. time). Our findings suggested that haze was positively related to the money donation intention, while it negatively related to the time donation intention. The two directions were opposite, so we should discuss the effect of haze on money and time separately.

In study 1, the result in money donation intention showed that people in haze weather condition would like to donate more money than in good weather condition. The result in time donation intention showed people in haze weather condition would like to donate less time than in good weather condition. Both results indicated that people devalue money and overvalue time in haze weather condition. We will explore the underlying mechanism of the different attitudes toward money and time in the next study.

Furthermore, we found a significant interaction effect between weather condition and donation intention type. Previous literature shows that people in air pollution weather feel more anxious, angry, and depressed (Zeidner and Shechter, 1988; Chattopadhyay et al., 1995), and our results confirmed it. We found that people experienced more negative emotion in haze weather compared with in good weather condition (*M*_*haze*_ = 3.57 vs. *M*_*good*_ = 5.31; *F*(1,108) = 41.43, *p* = 0.000, η^2^ = 0.28). However, the interaction effect between weather condition and donation intention type was still reliable after we controlled the emotion aroused by different weather conditions and the demographic variables.

## Experiment 2: The Mediation Effect of Mortality Salience

In study 1, we examined the relationship between haze and donation intention, and examined the moderation effect of donation type. Two aims guide our next study. First, we conducted study 2 to test the moderation effect again and make it more reliable. Second, more importantly, in this study, we measured mortality salience as the underlying mechanism to test the mediation effect.

### Research Method

#### Participants and Design

Study 2 is a 2 (weather condition: haze vs. good) × 2 (donation intention type: money vs. time) mixed subjects design as study 1, with donation intention (money vs. time) being the within group factor. The dependent variables are the donation intention, the amount of money donation, and the behavioral measurement for time donation (give email address or not). In this study, 101 participants (*M*_*age*_ = 28.78, SD = 7.06; 59.4% female) were recruited.

#### Materials and Procedures

All participants were asked to take part in two short and unrelated studies. The first one was weather perception survey and the second was donation survey. In the weather perception survey, half of the participants were randomly assigned to haze condition and the other half to good weather condition. And then all participants were instructed to involve the same donation survey task. So far, the procedures, stimulus, and the measurement of dependent variables were kept the same as study 1. After that, we measured mortality salience using three items (Conte et al., 1982; Abdel-Khalek, 1998). They were “In that weather condition, I have thought of death/I am afraid of death/I am afraid of losing life” (α = 0.87). Participants were asked to report their agreement on these statements (1 = strongly disagree, 7 = strongly agree).

### Data Analysis

Study 2 mainly investigated the impact of haze weather condition on donation and the mediation effect of mortality salience. Specially, people in haze condition, compared with good weather condition, would like to donate more money and less time, and this effect is driven by mortality salience. Therefore, this study first used a MANOVA to analyze the main effect of haze on donation, and then we adopt a repeated measure ANOVA to examine the moderation effect of donation type. Moreover, we conduct a series of regression models to check the mediation effect of mortality salience, and bootstrapping analysis is also tested to deepen our understanding of the underlying process.

### Results

#### Manipulation Checks

The one-way ANOVA analysis showed that people in haze condition reported more severe air pollution than good weather condition (*M*_*haze*_ = 5.98 vs. *M*_*good*_ = 2.43; *F*(1,100) = 368.18, *p* = 0.000, η^2^ = 0.79).

#### Hypothesis Testing

**FIGURE 2:**
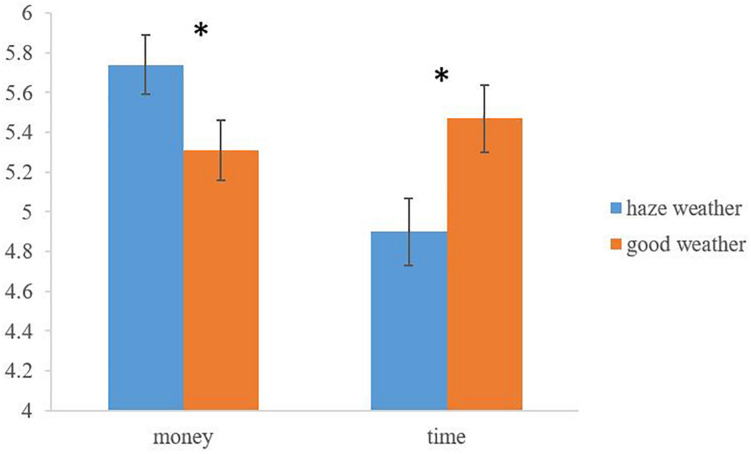
Moderation effect of donation type. *Significant at *p* < 0.05.

We first conducted ANOVA to examine the effect of haze on mortality salience. The data showed that compared with good weather, people in haze weather condition reported higher level of mortality salience (*M*_*haze*_ = 5.01 vs. *M*_*good*_ = 2.28; *F*(1,100) = 132.24, *p* = 0.000, η^2^ = 0.57), which supported our Hypothesis 1. We next conducted MANOVA to test the effect of haze on donation intention and behavior. The results revealed that there were significant differences between haze condition and good weather condition in intention to donate money (*F*(1,100) = 3.90, *p* = 0.05, η^2^ = 0.04) and intention to donate time (*F*(1,100) = 5.85, *p* = 0.017, η^2^ = 0.06). There was no difference between two conditions in the amount of donating money (*F*(1,100) = 0.17, *p* = 0.684). We got the same results after controlling demographic variables. When the behavioral measurement for time donation was the dependent variable, χ^2^ analysis did not reveal significant results as study 1 (χ^2^(1) = 2.31, *p* = 0.161). Besides, the moderated role of donation type was validated by repeated ANOVA. Results suggested that there was a significant interaction effect between haze and donation type (*F*(1,99) = 10.55, *p* = 0.002, η^2^ = 0.10; see Figure 2). Specifically, people in haze condition would donate more money (*M*_*haze*_ = 5.74 vs. *M*_*good*_ = 5.31; *F*(1,99) = 3.90, *p* = 0.05, η^2^ = 0.04) and less time (*M*_*haze*_ = 4.90 vs. *M*_*good*_ = 5.47; *F*(1,99) = 5.85, *p* = 0.017, η^2^ = 0.06; see Figure 2) compared with people in good weather condition.

**FIGURE 3:**
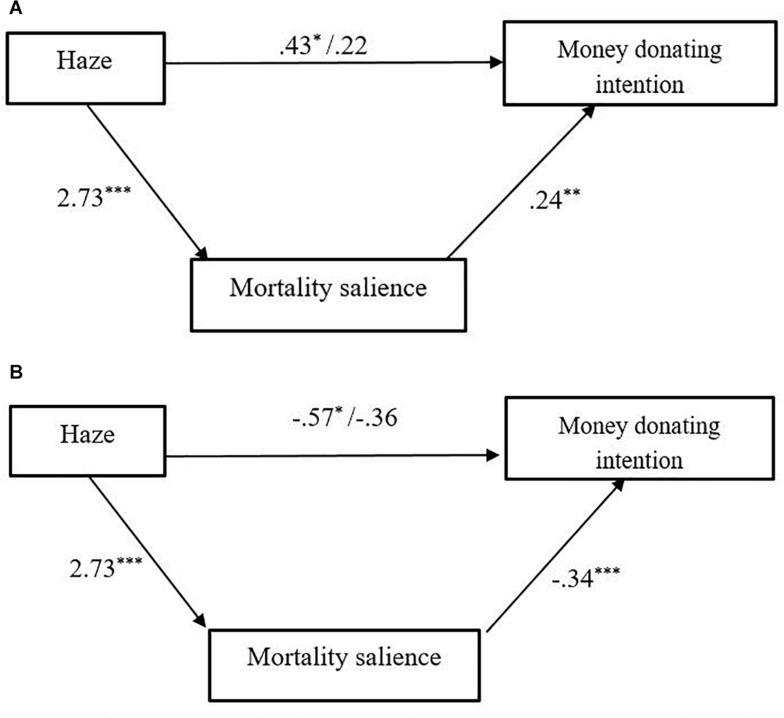
**(A)** Mediating effect of mortality salience on money donating intension. *p < 0.05, **p < 0.01, **p < 0.001. **(B)** Mediating effect of mortality salience on time donating intension *p < 0.05, **p < 0.01, **p < 0.001.

To test the mediation effect of mortality salience, we conducted a series of linear regressions. First, to examine the positive role of mortality salience on donating money, we ran regressions with intention of donating money as the outcome variable. Results showed that haze had a positive effect on mortality salience (*b* = 2.73, *t* = 11.50, *p* = 0.000), as well as money donation (*b* = 0.43, *t* = 1.97, *p* = 0.05), which suggested that haze could increase an individual’s mortality salience and intention on money donation. Mortality salience could positively predict intention of donating money (*b* = 0.19, *t* = 3.29, *p* = 0.001). When we included both haze and mortality salience into the regression model, the effect of mortality salience on money donating intention still was significant (*b* = 0.24, *t* = 2.67, *p* = 0.009; see Figure 3A) whereas the effect of haze on money donating intention became non-significant (*t* < 0.68, *p* = 0.495). To enhance our confidence of the mediating role of mortality salience on money donating intention, bootstrapping analysis was conducted (Hayes, 2013). The results confirmed that the indirect effect of haze on money donating intention via mortality salience was significant (95% CI 0.156, 1.160). Similarly, another series of linear regressions with time donating intention showed that both haze (*b* = −0.57, *t* = 2.42, *p* = 0.017) and mortality salience (*b* = −0.27, *t* = −4.31, *p* = 0.000) had a negative effect on time donation, and when they both were included into the regression function, only the effect of mortality salience remained significant (*b* = −0.34, *t* = −3.62, *p* = 0.000; see Figure 3B). Furthermore, the bootstrapping analysis also revealed the significant mediating role of mortality salience on time donating intention (95% CI −1.531, −0.476).

### Discussion

In study 2, there was no main effect of haze on the averaged donation intention, and significant effects appeared when we differentiated the donation intention into different types: money and time. The result in money donation intention showed that people in haze weather condition were more likely to donate more money compared with in good weather condition; the result in time donation intention showed people in haze weather condition would like to donate less time than in good weather condition. Both results indicated that people devalue money and overvalue time in haze weather condition.

In study 2, we tested mortality salience as the potential mediation variable to explain the different attitudes toward money and time in haze weather condition. Our results confirmed the hypotheses that mortality salience could mediate the interaction effect between haze (or not) and donation type. In haze condition, the thought of mortality was more salient in people’s minds. People valued time and devalued money so that in haze condition, the donation intention with time was lower and donation intention with money was higher compared with in good weather condition. Our findings were consistent with the previous literature about mortality salience. Previous studies found that people who were manipulated in mortality salience condition were more likely to spend more money to fulfill their materialism needs (Kasser and Sheldon, 2000), donate more money for pro-environmental campaign (Wade-Benzoni et al., 2012), and are more aware of the scarcity of time and value time more (King et al., 2009). In sum, we also found the interaction effect between haze and donation type. This effect was only verified when donation intention was the dependent variable, and it was not significant when the amount of donating money and behavioral measurement for time donation were the dependent variables. These findings replicated the results of study 1. More importantly, we tested the role of mortality salience. A series of regression models and bootstrapping analysis confirmed its mediation effect. Study 2 gave more support to understand the process of haze effect.

## General Discussion

### The Influence of Haze on Decision-Making

Little literature has investigated how haze weather influences donation behavior in psychology field. Except for this theoretical gap, more attention should be paid on this topic considering haze hits Southeast Asia frequently in recent years and it became a high concern for the policy-makers and the public (Zhang et al., 2014). The current research shows that haze could induce mortality salience and then make people devalue money and overvalue time, so that people would be more likely to donate money and less likely to donate time compared with control condition. We conducted two studies to test these propositions. In study 1, the data show that donation type (money vs. time) would be a core factor that can moderate the effect of haze on donation. Further, in study 2, the findings replicated the results of study 1, and verified the mediating role of mortality salience. Two studies provide the convergent evidence for our hypotheses. Therefore, our research can deepen our understanding on how negative environment (haze) can influence decision-making behavior.

### The Effect of Haze on Donation Intention

We provide initial insights into how haze has an impact on donation intention. Haze has been discussed in biological, environmental science, and sociological fields (Huang et al., 2014; Wang and He, 2014; Calderon-Garciduenas et al., 2015; Han et al., 2017). Based on the previous literature, we argued that people would tend to donate more money and less time on haze weather condition. However, we only found the significant effect of haze on donation intention. As for our other two dependent variables, the amount of money donation and the behavioral measurement for time donation (whether they would like to leave their email addresses to be a volunteer in the future which would take some time), there was no significant difference compared with control condition. Therefore, our findings have gone some way toward enhancing our understanding of this novel topic.

### The Effect of Mortality Salience on Money vs. Time

This research also provides a deeper insight into mortality salience and how it can influence people’s attitude on money and time. Previous literature about mortality salience already investigated the antecedents of mortality salience and suggested that diseases, disasters, and even subliminal prime about “death” can make mortality more salient (Arndt et al., 1997; Fritsche et al., 2010; White et al., 2013). Our results show that as a negative environment, haze also can induce mortality salience and bring people’s thought about death. Besides, we confirmed that people hold different attitudes on money and time when the mortality is salient in their mind. This finding is consistent with previous literature on this topic in which people would spend more money to fulfill materialism needs (Kasser and Sheldon, 2000), donate more money for pro-environmental campaign (Wade-Benzoni et al., 2012), and are more aware of the scarcity of time (King et al., 2009) when they perceive mortality salience. Our research thus contributes to the mortality salience literature.

### The Role of Donation Context

One of the limitations and the potential research direction is the role of donation context. In our two studies, the donation context both are about lung cancer, which is actually related to haze weather or air pollution. We propose that relatedness of donation context could be another moderator. That is, the effect of haze on donation type only exists when the donation context is related to air pollution, such as donation toward a lung cancer patient, but this effect may go away if the context is unrelated to air pollution or haze, such as donation to poor students. Further research is required to examine this possibility.

## Data Availability Statement

The datasets generated for this study are available on request to the corresponding author.

## Ethics Statement

Ethical review and approval was not required for the study on human participants in accordance with the local legislation and institutional requirements. Written informed consent for participation was not required for this study in accordance with the national legislation and the institutional requirements.

## Author Contributions

SL and GF designed the study and oversaw all aspects of the study implementation and data collection. SL, JY, and JW wrote the first manuscript. SL and JY performed and managed the experiments procedures. JY and JW contributed to the experimental materials. GF reviewed the manuscript. All authors made critical revisions to the final draft and agreed to be accountable for all aspects of the work in ensuring that questions related to the accuracy or integrity of any part of the work are appropriately investigated and resolved.

## Conflict of Interest

JY was employed by the company China Construction Bank. The remaining authors declare that the research was conducted in the absence of any commercial or financial relationships that could be construed as a potential conflict of interest.
